# The Carotid and Middle cerebral artery Occlusion Surgery Study (CMOSS): a study protocol for a randomised controlled trial

**DOI:** 10.1186/s13063-016-1600-1

**Published:** 2016-11-16

**Authors:** Yan Ma, Yuxiang Gu, Xiaoguang Tong, Jiyue Wang, Dong Kuai, Donghai Wang, Jun Ren, Lian Duan, Aili Maimaiti, Yiling Cai, Yujie Huang, Xiaojian Wang, Yi Cao, Chao You, Jiasheng Yu, Liqun Jiao, Feng Ling

**Affiliations:** 1 Department of Neurosurgery, Xuanwu Hospital, Capital Medical University, No. 45 Changchun Street, Beijing, China; 2 Department of Neurosurgery, Huashan Hospital, Fu Dan University, No. 12 Mid Wulumuqi Road, Shanghai, 200040 China; 3 Department of Neurosurgery, Huanhu Hospital, No. 6 Jizhao Road, Tianjin, 300350 China; 4 Department of Neurosurgery, Brain Hospital, No. 45 Huashan Road, LiaoCheng, Shandong 252000 China; 5 Department of Neurosurgery, The 1st Affiliated Hospital of ShanXi Medical University, No. 85 South Jiefang Road, Taiyuan, Shanxi 030001 China; 6 Department of Neurosurgery, Qilu Hospital of Shandong University, No. 107 West Wenhua Road, Jinan, Shandong 250012 China; 7 Department of Neurosurgery, The 2nd Affiliated Hospital of LanZhou University, No. 80 Cuiyingmen Road, Lanzhou, Gansu 730030 China; 8 Department of Neurosurgery, The 307 Hospital of PLA, No. 8 Dongda Street, Beijing, 100071 China; 9 Department of Neurosurgery, The 1st Affiliated Hospital of XinJiang Medical University, No. 137 South Liyushan Road, Wulumuqi, Xinjiang Uyghur Autonomous Region 830054 China; 10 Department of Neurology, The 306 Hospital of PLA, No. 9 Anxiangbeili, Beijing, 100101 China; 11 Department of Neurosurgery, Drum Tower Hospital, Medical School of Nanjing University, No. 321 Zhongshan Road, Nanjing, Jiangsu 210008 China; 12 Department of Neurosurgery, The 1st Affiliated Hospital of Anhui Medical University, No. 218 Jixi Road, Hefei, Anhui 650101 China; 13 Department of Neurosurgery, The 2nd Affiliated Hospital of Kunming Medical University, No. 324 Dianmian Road, Kunming, Yunnan 610041 China; 14 Department of Neurosurgery, West China Hospital of Sichuan University, No. 37 Guoxue Street, Chengdu, Sichuan 610041 China; 15 Department of Neurosurgery, Tongji Hospital, Tongji Medical College, Huazhong University of Science and Technology, No. 1095 Jiefang Road, Wuhan, Hubei 430030 China

**Keywords:** Carotid artery, Middle cerebral artery, Occlusion, Haemodynamic, Bypass surgery, Ischaemic stroke, China

## Abstract

**Background:**

Patients with symptomatic internal carotid artery (ICA) or middle cerebral artery (MCA) occlusion with haemodynamic insufficiency are at high risk for recurrent stroke when treated medically.

**Methods:**

The Carotid or Middle cerebral artery Occlusion Surgery Study (CMOSS) trial is an ongoing, government-funded, prospective, multicentre, randomised controlled trial. The CMOSS will recruit 330 patients with symptomatic ICA or MCA occlusion (parallel design, 1:1 allocation ratio) and haemodynamic insufficiency. Participants will be allocated to best medical treatment alone or best medicine plus extracranial-intracranial (EC-IC) bypass surgery. The primary outcome events are all strokes or deaths occurring between randomisation and 30 days post operation or post randomisation and ipsilateral ischaemic stroke within 2 years. Recruitment will be finished by December 2016. All the patients will be followed for at least 2 years. The trial is scheduled to complete in 2019.

**Discussion:**

The CMOSS will test the hypothesis that EC-IC bypass surgery plus best medical therapy reduces subsequent ipsilateral ischaemic stroke in patients with symptomatic ICA or MCA occlusion and haemodynamic cerebral ischaemia. This manuscript outlines the rationale and the design of the study. CMOSS will allow for more critical reappraisal of the EC-IC bypass for selected patients in China.

**Trial registration:**

NCT01758614 with ClinicalTrials.gov. Registered on 24 December 2012.

## Background

Patients with symptoms of cerebral ischaemia associated with ipsilateral internal carotid artery (ICA) occlusion have an annual risk of 5–8% of recurrent ischemic stroke [1, 2]; patients with symptomatic middle cerebral artery (MCA) disease (including severe stenosis and occlusion) have an overall stroke risk of 12.5% per year (ipsilateral: 9.1%) [3]. The annual risks of recurrent ischaemic stroke in patients with symptomatic ICA or MCA occlusion have not obviously improved over recent years [4]. The proportion of recurrence with minor disabling consequences was rather high.

### The pathophysiology of carotid and middle cerebral artery occlusion

#### Collateral circulation

Compensation by a collateral circulation is usually considered important in the pathophysiology of cerebrovascular stenosis and occlusion. The Circle of Willis as the primary collaterals provides immediate diversion of cerebral blood flow (CBF) to ischaemic regions. The compensatory capacity of the primary collaterals depends on their vascular calibre and patency. The ophthalmic artery and leptomeningeal vessels constitute secondary collaterals, and also contribute ancillary collateral flow to distal segments of stenotic or occluded major cerebral arteries [5]. In a case of occlusion of the ICA or MCA over a chronic time course, the features of the secondary collaterals are related to the severity of haemodynamic alterations and metabolic and neuroendocrine mechanisms [5].

Patterns of collateral flow in chronic occlusive cerebrovascular disease are different between the ICA and the MCA [6, 7]. In patients with ICA occlusion, possible compensatory blood flow could be from the contralateral ICA via the anterior communicating artery (AComA) or from the vertebrobasilar system via the posterior communicating artery (PcomA). While anatomic studies show that about 1% of subjects have an absent AComA, 10% of subjects have an absent or hypoplastic proximal anterior cerebral artery and 30% of subjects have absence or hypoplasia of one or other PComA [8].

Because the MCA is located in the downstream of the Circle of Willis, the primary collaterals fail to compensate for the loss of blood flow perfusion; only pial or meningeal to pial collaterals are available in patients with MCA occlusion [6, 7]. However, the pial collaterals are not adequate to maintain normal cerebral haemodynamics. Thus, poor collateral development is considered a risk factor for stroke in patients with MCA occlusion [6, 7.]

Additionally, the development of a collateral circulation does not guarantee its function and its function may be related to age, comorbidities and the duration of ischaemia [9]. Haemodynamics is one of the most important factors [7].

#### Cerebral haemodynamics

The haemodynamic stroke is a kind of stroke subtype caused by local or systemic hypoperfusion. Occlusion of the ICA or MCA is the local factor that contributes to impaired haemodynamics although low blood pressure (BP) or volume might also play a systemic role [10]. According to cerebrovascular autoregulatory mechanisms, CBF could remain static during steady-state changes in cerebral perfusion pressures (CPPs) within a wide range by the occurrence of distal vasodilation. Vasomotor reactivity (VMR) or cerebral vasoreactivity (CVR) is used to describe this capability of cerebral resistance arterioles to change their calibre when CPPs decrease. Decreased VMR indicates preexisting vasodilation induced by ICA or MCA occlusion. When distal vasodilation becomes maximal and CPPs continue to decline, the brain tissue increases the oxygen extraction from the blood in order to maintain normal metabolism and function [11]. That is termed ‘misery perfusion’ or stage II haemodynamic compromise [10]. There is increasing evidence that cerebral haemodynamics play an important role in the long-term prognosis of patients with chronic ICA or MCA occlusion. Some investigators report that the annual risk of ipsilateral stroke is 32.7% in ICA occlusion patients with impaired VMR and poor collateral circulation [4]. Derdeyn et al. also demonstrated that in patients with MCA occlusion, the frequency of impaired haemodynamic compromise was high [12]. So patients in these situations might benefit from revascularisation surgery.

### Cerebral haemodynamic evaluation

Cerebral haemodynamic evaluation is the core factor for patients with ICA or MCA occlusion. Quantitative xenon-computed tomography-(CT) can provide exact data of regional CBF and, accompanied with the Diamox challenge test, can determine the VMR/CVR convincingly [13]. Xenon-CT with the Diamox challenge test has been utilised widely in Japan and the USA, while recently the spread of its usage across the world has been slowed down because of the current limitations in access of FDA.

[^15^O]H_2_O positron emission tomography (PET) not only provides quantitative CBF values, but also cerebral metabolism information. As the basis of the Carotid Occlusion Surgery Study (COSS) funded by the NIH (National Institutes of Health), the St. Louis Carotid Occlusion Study (STLCOS) was performed in order to evaluate the cerebral haemodynamics of patients with ICA occlusion systematically using PET [11]. Using a semiquantitative, count-based hemispheric oxygen extraction fraction (OEF) ratio method, STLCOS first identified the normal OEF ratio threshold from normal control subjects. The risk of all strokes was then found to be 6.0 times higher in patients with stage II haemodynamic failure (with increasing OEF) compared to normal OEF, and the risk of ipsilateral stroke was 7.3 times higher. Unfortunately, because different countries have different situations, [^15^O]H_2_O PET is still limited in many countries, especially in developing countries, because access to [^15^O] requires an on-site cyclotron.

With the spread of modern computed tomography (CT) scanners, the CT perfusion (CTP) technique is easily available in most institutes. Remarkable developments have been achieved in perfusion techniques even though measurement variability (precision) is in the range of 15–30% when region of interest (ROI)-based techniques are used [14]. According to the perfusion technique mechanism, several investigations have tried to identify the normal thresholds for interpreting measurements made with this technique. Waaijer et al. [15] identified that the least variability is located in the MCA territory. Calculation of CTP ratios of bilateral hemispheres may eliminate the variations caused by the selection of the arterial input function (AIF) and venous output function (VOF). If the ROIs drawn in bilateral hemispheres are symmetrical, CTP ratios will be helpful to eliminate the resulting measurement variability. The COSS investigators [16] also tried to compare CTP with [^15^O]H_2_O PET in patients with chronic ICA occlusion and found mean transit time (MTT) to be the best parameter, correlating with the count-based PET OEF ratios (slope = 0.124, intercept = 0.676, *R* ^2^ = 0.590, *p* < 0.001). CTP relative CBF (rCBF) compares favorably to PET relative CBF when processed using a dedicated AIF for each territory (*R* ^2^ = 0.796, *p* < 0.001). According to these results, a combination of decreased CBF ratio (symptomatic side/asymptomatic side) and increasing MTT could help to distinguish stage II haemodynamic failure.

## Methods

### Trial design

The Carotid or Middle cerebral artery Occlusion Surgery Study (CMOSS) trial in China is designed to compare the safety and efficacy of EC-IC bypass surgery with medical therapy in patients with symptomatic ICA or MCA occlusion (within 120 days). It is the subproject of the ‘Five-twelfth’ National Science and Technology Support Program funded by the Chinese government and is a randomised controlled trial (RCT) that plans to enroll 330 patients with equal randomisation to surgical and nonsurgical groups. The allocation ratio will be 1:1. The CMOSS trial will be conducted in 15 centres in China.

### Inclusion and exclusion criteria

#### Inclusion criteria

1. Age ranging between 18 and 65 years
2. Digital subtraction angiography (DSA) imaging studies demonstrating occlusion of a unilateral ICA or MCA
3. DSA studies demonstrating less than 50% stenosis of any other vessels especially the contralateral ICA, MCA or basilar artery
4. Modified Rankin Scale (mRS) score 0–2
5. A qualifying transient ischaemic attack (TIA) or ischaemic stroke in the territory of the occluded ICA or MCA must have occurred within the past 12 months
6. The most recent stoke occurred more than 3 weeks previously
7. The neurological deficit must have been stable for more than 1 month
8. No massive cerebral infarction (more than 50% of the MCA territory) demonstrable in a CT or magnetic resonance imaging (MRI) study
9. CTP demonstrates ‘misery perfusion’ (MTT more than 4 s and rCBF (symptomatic side/asymptomatic side) < 0.95)
10. Must be competent to give informed consent
11. Must be legally an adult
12. Must be geographically accessible and reliable for follow-up

#### Exclusion criteria

1. Other neurovascular disease (such as cerebral aneurysm or arteriovenous malformation) conditions likely to cause focal cerebral ischaemia
2. Known unstable angina or myocardial infarction within the last 6 months
3. Pregnant or perinatal stage women
4. Coagulopathy
5. Any diseases likely to lead to death within 2 years
6. Past history of EC-IC bypass surgery
7. Any contraindications or allergy to aspirin or clopidogrel
8. Any heart disease likely to cause cerebral ischaemia including prosthetic valve(s), infective endocarditis, atrial fibrillation, sick sinus syndrome, myxoma, and cardiomyopathy with an ejection fraction of less than 25%
9. Allergy to iodine or radiographic contrast media
10. Serum creatinine level > 3 mg/dl
11. Uncontrolled diabetes mellitus (fasting blood glucose level > 16.7 mmol/l)
12. Uncontrolled hypertension (systolic BP above 180 mmHg, diastolic BP above 110 mmHg)
13. Severe liver dysfunction (alanine transaminase (ALT) and/or aspartate transaminase (AST) more than three times the normal level)
14. Concurrent participation in any other experimental treatment trial
15. Any condition that in the surgeon’s judgment suggests the patient to be an unsuitable surgical candidate

### Intervention and follow-up

After an Informed Consent Form (ICF) has been signed, all the patients will be given best medical treatment including management of risk factors (elevated systolic BP, elevated low-density lipoprotein cholesterol level, diabetes mellitus, smoking, excess weight). Aspirin (100 mg per day) or clopidogrel (75 mg per day) will be prescribed to the patient and they will discontinue the use of any other antithrombotic therapy (specifically, oral anticoagulant agents) until the 30-day follow-up visit. After that, the experienced neurologists caring for the patient will determine the necessity of antithrombotic therapy.

Surgical intervention will be performed within 7 days after randomisation by attending neurosurgeons who have been certified as chief surgeon in at least 15 consecutive previous EC-IC bypass surgeries. Their surgical record of anastomosis graft patency is greater than 95% and the perioperative stroke and death rate is less than 10%. Standard end-to-side anastomosis of the superficial temporal artery (STA) to the M4 segment (MCA) will be utilised. If the STA is unsuitable, the occipital artery may be used.

All the patients will be followed up at the neurological outpatient clinic on four occasions (at 30 days, 6, 12, and 24 months). Scheduled follow-up examinations are summarised in Table 1. If a potential endpoint event occurs, patients will be examined by neurologists within 24 h. If a stroke is suspected, MRI or CT will be performed as soon as possible.

**Table 1:** Follow-up schedule

	Baseline	7 days	30 days	6 months	12 months	24 months
Neurological function evaluation						
NIHSS	✓	✓	✓	✓	✓	✓
mRS	✓	✓	✓	✓	✓	✓
BI	✓	✓	✓	✓	✓	✓
Neuro-imaging evaluation						
Brain CT/MRI	✓DWI	✓	✓	✓	✓	✓
Cerebrovascular CTA/MRA					✓	
DSA	✓	✓^a^			✓^b^	✓
CTP	✓	✓^a^		✓	✓	✓
Medication						
Current medication	✓	✓	✓	✓	✓	✓
Medication compliance		✓	✓	✓	✓	✓
Adverse events	✓	✓	✓	✓	✓	✓
Physical examination	✓	✓	✓	✓	✓	✓
Vital signs	✓	✓	✓	✓	✓	✓
Laboratory tests	✓	✓^a^		✓	✓	✓
Pregnancy test	✓					✓
Electrocardiogram	✓					✓
Chest X-ray	✓					✓

^a^Bypass surgical group only; ^b^If necessary; *CT* computed tomography, *CTA* computed tomography angiography, *CTP* computed tomography perfusion, *DSA* digital subtraction angiography, *DWI* diffusion-weighted imaging, *MRA* magnetic resonance angiography, *MRI* magnetic resonance imaging, *mRS* modified Rankin scale, *NIHSS* National Institutes of Health Stroke Scale

### Outcome events and study measures

The primary outcome events will be defined as all strokes or deaths occurring between randomisation and the 30-day postoperative time point (for the nonsurgical group, this will be defined as all strokes or deaths occurring within 30 days post randomisation) and ipsilateral ischaemic stroke within 2 years post randomisation. A stroke is defined as rapid loss of neurological function due to ischaemia or a haemorrhage. Ipsilateral ischaemic stroke is further defined as the clinical diagnosis of a focal neurological deficit due to cerebral ischaemia that is clinically localisable within the territory of the symptomatic occluded ICA or MCA and that lasts for more than 24 h. CT or MRI scanning is necessary to identify the stroke.

Secondary outcome events include all strokes, disabling or fatal strokes and deaths between 30 days and 2 years post randomisation; severe TIA; complications associated with the surgical procedures; mRS score, National Institutes of Health Stroke Scale score (NIHSS) and Barthel Index assessment within 2 years; and anastomosis patency and haemodynamic changes within 2 years.

### Sample size and randomisation

Sample size assumes that the true primary outcome rates will be 28% in the medical treatment group [17–19] and in the surgical group there will be 50% relative risk reduction. This means that the 2-year postoperative ipsilateral stroke or death rate was assumed to be 14% in the surgical group. This would require 264 (132 per group) participants to provide valid data to ascertain this endpoint of the study (power of 80% and two-tailed alpha of 5% significance level). Further anticipation includes as many as 20% loss to follow-up or early withdrawal, which would require that 330 participants enroll in the study (165 per group).

A Central Imaging Evaluation Committee (CMEC) will review all the image data of every candidate before randomisation. Subsequently, when a local investigator receives the permitted information from CMEC, IVRS (Interactive Voice Response System, Clinicalsoft Company Limited) will be used for randomisation. Patients will be randomised (in a 1:1 ratio, with a random permuted block design (block size 2, 4, or 6) stratified by clinical site) to medical treatment alone or medical treatment plus bypass surgery. Patients, clinicians, and investigators are aware of treatment assignment.

## Discussion

### Review of COSS

The recently published COSS [20] failed to show a benefit of EC-IC bypass surgery over medical therapy in patients with symptomatic haemodynamically significant carotid occlusion. Since then different controversies [21, 22] have been raised regarding several aspects of the study including the study population, the surgeons’ qualifications, and haemodynamic evaluation.

In the COSS protocol [23], the primary inclusion population is a patient demonstrating occlusion of the unilateral ICA with the contralateral ICA demonstrating less than 50% stenosis. While in the final report, only 18% of patients demonstrated a contralateral ICA stenosis of more than 50% because of enrolment problems [20]. As we know, the COSS utilised the OEF ratio by PET as the criterion of haemodynamic evaluation. However, bilateral ICA lesions will disturb this ratio and will even disturb the identification of the subgroup of patients with haemodynamic insufficiency.

As an interventional trial, the COSS should ensure the certification of the experienced surgeons. However, for expanding the number of centres and enhancing recruitment, the COSS made some concessions on the surgeons’ training and certification; the 15% postoperative event rate is not the best that can be achieved. In a recently published article about the analysis of mechanisms of perioperative ischaemic stroke by the COSS investigators [24], they found that the temporary occlusion time of the MCA during the anastomotic procedure (54.3 ± 23.5 min) was no different from the occlusion time in those surgical patients who did not sustain a perioperative stroke (45.4 ± 24.2 min, *p* = 0.2). Under the strict intraoperative management protocol and within the temporary vessel occlusion time, which ranged from between 25 and 42 min (mean 33.6 ± 7 minutes) as used in the study by Horn et al. [25], the postoperative ischaemic stroke rate should be less than 10%. This discrepancy would suggest that these underlying assumptions warrant further exploration and more rigorous research.

The COSS Kaplan-Meier curve demonstrated a cross-over benefiting surgery along with follow-up continuation. But, in fact the COSS was terminated early for ‘futility’. However, if all the enrolled patients had completed the follow-up interview the study might have had adequate power to show a significant benefit from surgery by 5 years [26].

### Rationale of CMOSS

The CMOSS is the first RCT sponsored by a developing country to evaluate the efficacy and safety of EC-IC bypass surgery. It is also the first RCT to evaluate the cerebrovascular haemodynamics by CTP in patients with ICA or MCA occlusion. It is well-known that intracranial arterial stenosis or occlusion in Asia is much more common than in Europe or North America. This could greatly encourage patient enrolment. The CMOSS also selects more experienced neurosurgeons to work in the trial to minimise perioperative complications.

For the sample size calculation, we consulted the figures for stroke risk from MCA occlusion, which is different from ICA occlusion. In 1996, Yamauchi et al. [17] investigated patients with symptomatic ICA or MCA occlusive diseases. They found that the incidence of ipsilateral ischaemic strokes in patients with increased OEF and normal OEF was 57.1% and 6.0%, respectively. (They also suggested that patients with increased OEF had decreased CBF and CBF/CBV values.) Kuroda et al. [19] reported that the annual risks of total and ipsilateral stroke in patients with ICA or MCA occlusion and decreased rCBF and relative cerebral vasoreactivity (rCVR) were 35.6% and 23.7%, respectively. Actually, the total annual risk of ipsilateral stroke in patients with decreased rCBF only was 26.1%. In 2002, Ogasawara et al. [18] presented a study to prospectively evaluate the relationships between cerebral haemodynamics and outcome of patients with symptomatic major cerebral artery occlusion (including ICA and MCA) by quantitative measurement of CBF using ^133^Xe and single photon emission tomography (SPECT). During the follow-up, the recurrence rate of stroke was 34.7% in patients with reduced rCVR at entry and less than 6% in patients with normal rCVR. All these data indicate that the annual risk of stroke is relatively higher in MCA than in ICA occlusion. Recently, aggressive medical management has been more widely used, and according to the results of a follow-up by our centre over the last 3 years, the risk of stroke in ICA or MCA occlusion is about 28%, which conforms to the data mentioned. Compared with the medical treatment group (28%), a further 50% reduction in the surgical treatment group is considered to be clinically significant; that is 14%. Regarding perioperative morbidity, in the COSS [20] the perioperative rate for ipsilateral ischemic stroke was 14.4%, while the rate for the primary end point was 21.0%. This indicates that the stroke rate between 30 days to 2 years was about 7%. In the CMOSS, we have much stricter licensing of neurosurgeons; attending neurosurgeons are certified by their roles as chief surgeon in at least 15 consecutive previous EC-IC bypass surgeries. Using this level of expertise means that in China we can achieve an anastomosis graft patency rate of greater than 95% and a perioperative stroke and death rate of less than 10%.

In addition, because of the continuity of the ‘Five-twelfth’ National Science and Technology Support Program, the follow-up for these patients could prolonged to more than 2 years. This could reveal more information after analysis of the ‘cross-over curves’ in the COSS.

### Trial status

The CMOSS trial is currently recruiting participants.

## Abbreviations

AComA: Anterior communicating artery
ALT: Alanine transaminase
AST: Aspartate transaminase
CBF: Cerebral blood flow
CPP: Cerebral perfusion pressure
CTP: Computed tomography perfusion
CVR: Cerebral vasoreactivity
DSA: Digital subtraction angiography
EC-IC: Extracranial-intracranial
ICA: Internal carotid artery
ICF: Informed Consent Forum
MCA: Middle cerebral artery
mRS: Modified Rankin Scale
MTT: Mean transient time
NIHSS: National Institutes of Health Stroke Scale score
OEF: Oxygen extraction fraction
RCT: Randomised controlled trial
VMR: Vasomotor reactivity
